# Case Report: The crucial role of contrast-enhanced ultrasound and intraoperative ultrasound in diagnosing pediatric pancreatoblastoma

**DOI:** 10.3389/fonc.2025.1610618

**Published:** 2025-11-19

**Authors:** Xiangfeng Zeng, Hualin Yan, Jiali Yang, Bo Xiang, Juxian Liu

**Affiliations:** West China Hospital, Sichuan University, Chengdu, China

**Keywords:** pancreatoblastoma, ultrasound, contrast-enhanced ultrasound, intraoperative ultrasound, pediatrics, case report

## Abstract

Pancreatoblastoma (PB) is a rare malignant neoplasm of the pancreas, primarily affecting children. While some reports have described the imaging characteristics of PB, detailed descriptions of its ultrasound (US) and contrast-enhanced ultrasound (CEUS) features in children are limited. We reported two cases of PB admitted to our hospital with detailed ultrasonographic features. The first case involved a 14-year-old girl who presented with intermittent, unexplained epigastric pain. CEUS revealed a hypoechoic mass with heterogeneous hyperenhancement in the pancreatic head. She underwent pancreaticoduodenectomy and remains disease-free to date. The second case was a 4-year-old boy with a palpable, unexplained mass in the right upper abdomen. US identified a well-defined, heterogeneous mass in the epigastric region with internal point-like hyperechoic areas. The intraoperative US showed portal vein cancer thrombus. He underwent tumor resection along with reconstruction of the portal and superior mesenteric veins. He subsequently received chemotherapy and remained disease-free to date.

## Introduction

Pancreatoblastoma (PB) is one of the most common malignant pancreatic neoplasms in children (1, 2). It is typically a slow-growing tumor with non-specific clinical presentations, often resulting in delayed diagnosis until the disease has reached an advanced stage (3). Since its first description by Frable et al. (4), several reports have discussed the imaging features of PB; however, detailed accounts of its ultrasonic characteristics remain rare. In this report, we present two cases of pediatric PB, providing a comprehensive analysis of their ultrasonographic features and emphasized the role of CEUS and intraoperative ultrasound.

## Cases presentation

### Case 1

A 14-year-old girl presented with unprovoked intermittent epigastric pain for the past 6 months. She also reported occasional episodes of nausea and vomiting. Abdominal palpation revealed no tenderness, rebound tenderness, or muscle tension. No palpable abdominal masses were detected. Tumor markers, including carcinoembryonic antigen (CEA), carbohydrate antigen 125 (CA125), and carbohydrate antigen 19-9 (CA19-9), were not significantly elevated. However, alpha-fetoprotein (AFP) was elevated at 395 ng/mL.

**Figure 1:**
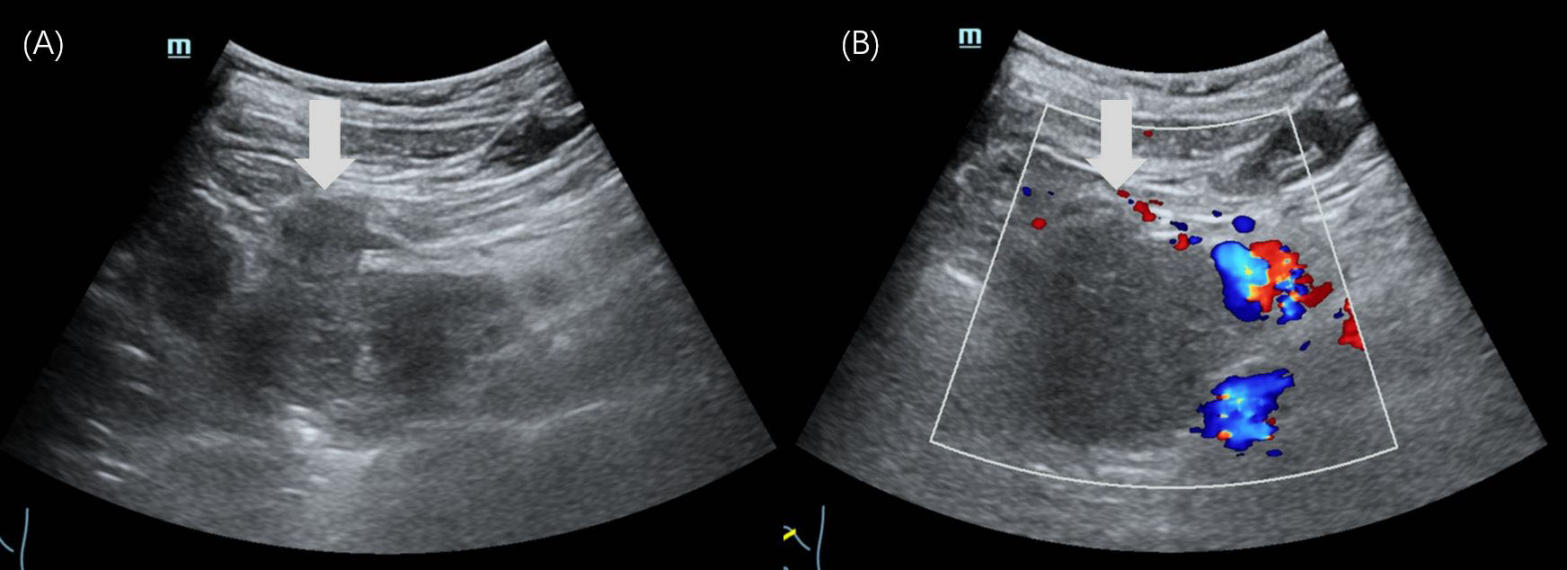
Ultrasound images of case 1. **(A)** The gray-scale US showed a 5.6×4.8×6.3cm irregular-shaped, indistinct-bounded, hypoechoic mass (arrow) in the pancreatic head. **(B)** The color Doppler imaging showed no obvious signal of blood flow within the mass (arrow).

**Figure 2:**
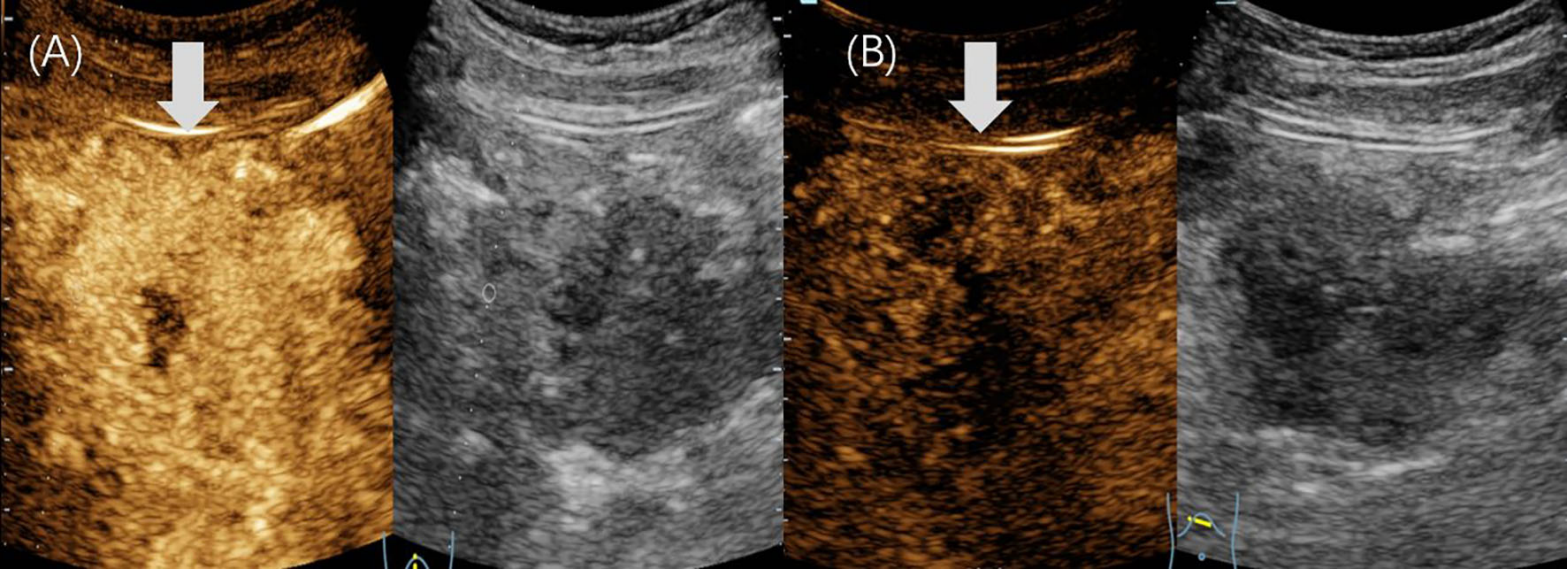
Contrast-enhanced ultrasound images of case 1. **(A)** In the arterial phase, the mass showed heterogeneous hyperenhancement (arrow). **(B)** The mass showed hypoenhancement (arrow) in the venous phase.

Abdominal US revealed an irregular, hypoechoic mass in the pancreatic head, measuring 5.6×4.8×6.3 cm, with indistinct margins and no obvious blood flow signals (**Figure 1**). The patient underwent CEUS with the patient’s consent for further diagnosis. A 1.2-mL US contrast agent SonoVue (Bracco, Milan, Italy) suspension was injected as a bolus (injection time <3 s) through the left cubital vein followed by a flush with 5 mL saline. The mass exhibits arterial-phase hyperenhancement of its solid components, demonstrating greater conspicuity than surrounding pancreatic parenchyma, with persistent non-enhancing necrotic regions (**Figure 2A**). Venous-phase imaging reveals rapid contrast washout in the solid components, resulting in hypoenhancement relative to pancreatic tissue, whereas necrotic areas maintain their avascular characteristics (**Figure 2B**). Contrast-enhanced computed tomography (CECT) showed a 6.0×4.5 cm soft tissue density tumor with indistinct margins in the head of the pancreas, exhibiting heterogeneous hyperenhancement and a hypodense necrotic area.

Based on the imaging findings, laboratory results, and the incidence of pancreatic tumors, the clinical diagnosis was pancreatoblastoma. She underwent pancreaticoduodenectomy, cholecystectomy, and resection and reconstruction of the portal and superior mesenteric veins. Intraoperative examination revealed a firm pancreas with marked inflammatory edema of the gland and surrounding tissues, whereas a hard, multinodular mass was identified in the pancreatic head and the uncinate process exhibited cystic-necrotic changes. Intraoperative US findings were consistent with the preoperative observations, with no new positive findings identified. Moreover, the clinical diagnosis was later confirmed by postoperative pathology. Immunohistochemical staining results were as follows: β-catenin (−), CK 8/18 (+), CgA (+), E-C 99 (+), GPC-3 (+). At the 23-month follow-up, there was no evidence of disease recurrence.

### Case 2

A 4-year-old boy presented to our hospital with an unexplained mass in the right upper abdomen for 1 week. Since the onset of the condition, he did not report any abdominal pain, nausea, vomiting, fever, or jaundice. A computed tomography (CT) scan conducted at a local hospital revealed a mass in the upper abdomen, initially suspected to be a right adrenal neuroblastoma.

**Figure 3:**
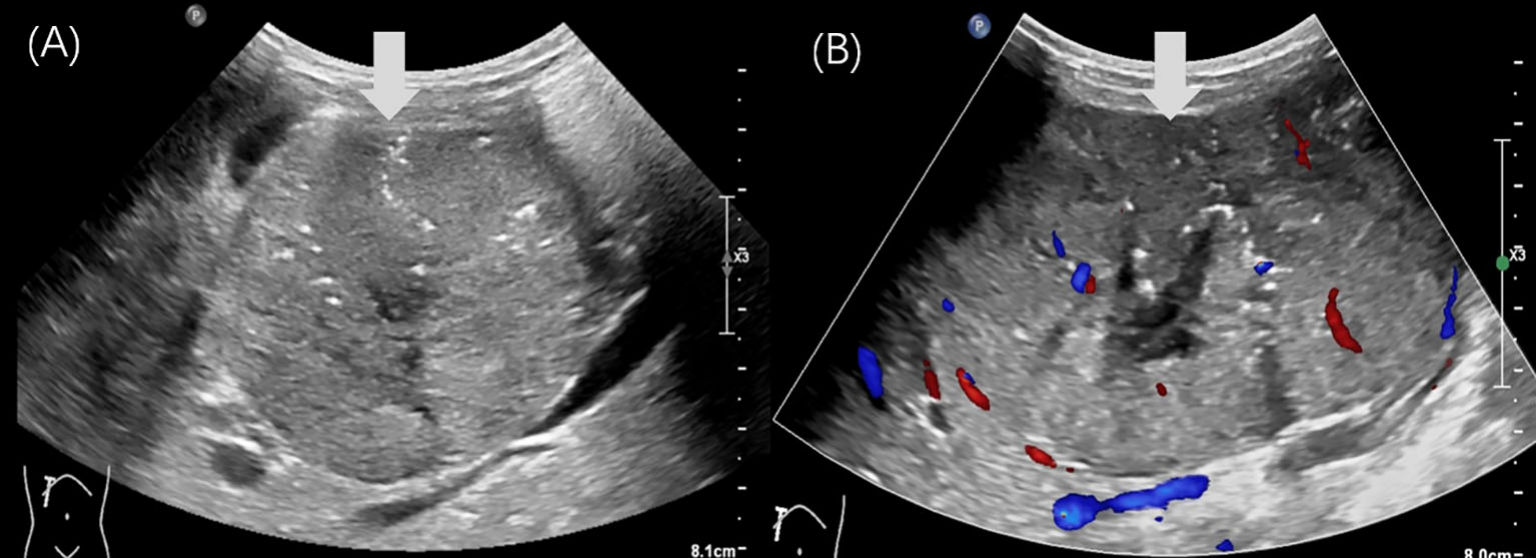
Ultrasound images of case 2. **(A, B)** The gray-scale US revealed a regular-shaped, heterogeneous, epigastric mass (arrow) with internal point-like hyperechoic, measuring 10.6×7.1cm in diameter. **(B)** The color Doppler imaging showed that a dotted linear blood flow (arrow) was visible within it.

On examination, a firm, non-tender mass measuring approximately 8 × 10 cm was palpable in the right upper abdomen, with limited mobility. Tumor markers, including CEA and CA19-9, were not significantly elevated. However, both AFP and neuron-specific enolase (NSE) levels were elevated (AFP: 1210 ng/mL, NSE: 51.2 ng/mL). Abdominal US revealed a well-defined, heterogeneous mass in the epigastrium, measuring 10.6 × 7.1 cm, with internal point-like hyperechoic regions. Dotted linear blood flow was detected within the mass (**Figure 3**). No enlarged lymph nodes were observed in the abdominal cavity. Additionally, CECT revealed a mixed-density mass in the hepatogastric space, measuring approximately 10.5 × 6.5 cm, with low-density areas and scattered calcifications. In the arterial phase, the mass exhibited heterogeneous hyperenhancement.

**Figure 4:**
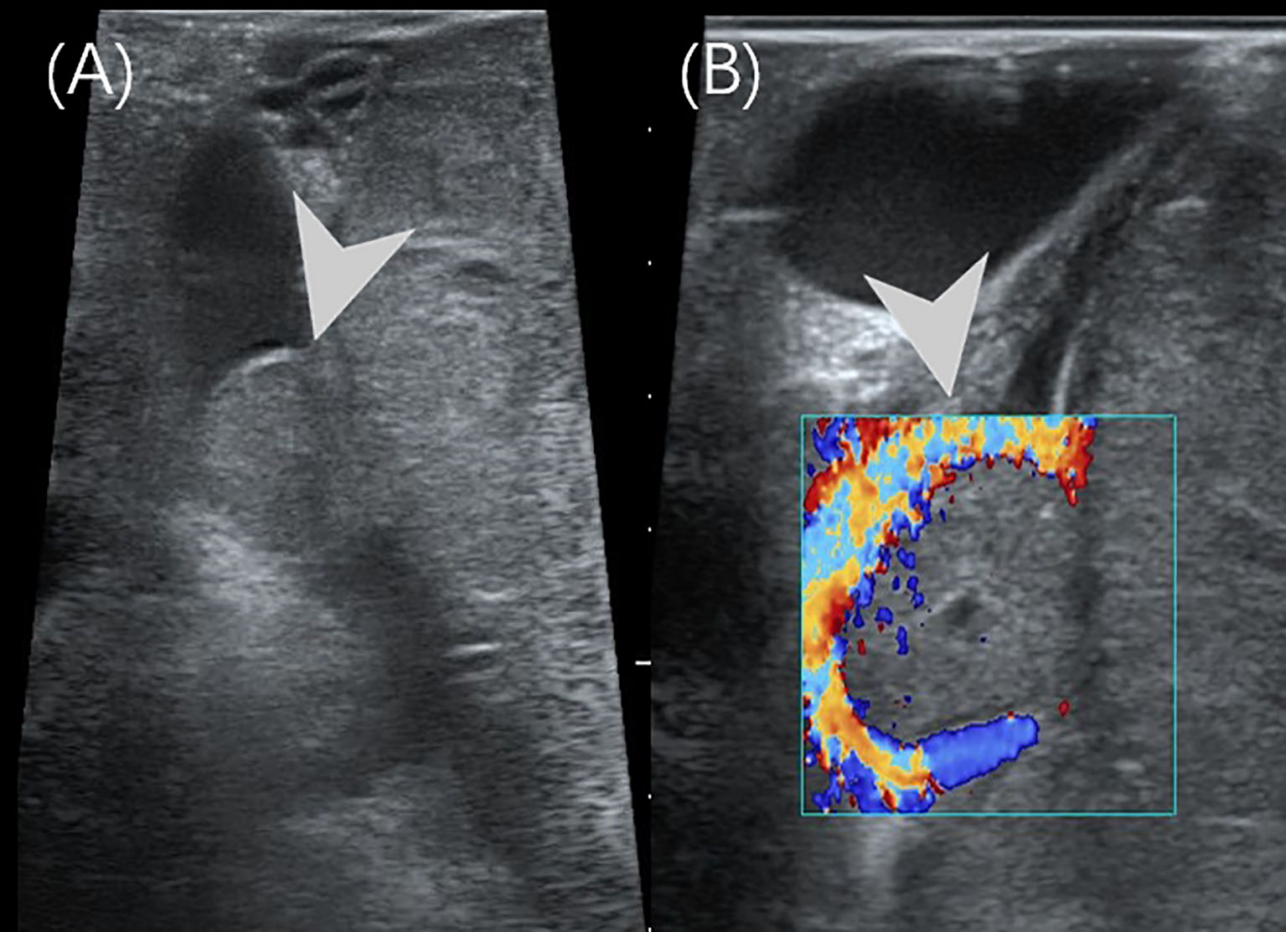
Intraoperative US of case 2. **(A)** The intraoperative US showed a 1.5×1.0cm hypoechoic nodules (arrowhead) in the main trunk of the portal vein. **(B)** The color Doppler imaging showed a filling defect in the main trunk of the portal vein.

Based on the imaging findings and laboratory results, the patient was clinically diagnosed with a retroperitoneal malignant tumor. Laparotomy revealed a large, cystic, and poorly mobile cystic-solid tumor in the retroperitoneal space. The mass demonstrated a vascular-rich surface, displaced the duodenum and hepatoduodenal ligament anteriorly, and invaded surrounding structures. It also encased the celiac trunk, portal vein, and its tributaries, compressing adjacent organs. The intraoperative US detected cancer thrombus in the portal vein (**Figure 4**), whereas no thrombus was detected in the inferior vena cava. Tumor resection was performed along with reconstruction of the portal and superior mesenteric veins. Postoperative histopathology revealed a round cell tumor with rosette formations under microscopic examination. Immunohistochemical staining showed the following results: β-catenin+ (nuclear staining of some cells), cytokeratin+, CK 19+, CD10+, CD99+, and Ki-67+ at 70%. These findings confirmed the diagnosis of pancreatoblastoma. The patient subsequently received chemotherapy. At the 21-month follow-up, there were no signs of tumor recurrence.

## Discussion

PB, also known as infantile pancreatic carcinoma, is an extremely rare pancreatic tumor in childhood, accounting for approximately 0.5% of pancreatic non-endocrine tumors (5). The term “pancreatoblastoma” was first introduced by Horie et al. in 1977 (6). PB predominantly affects young children, with a median age of 5 years (7). Boys are affected approximately twice as often as girls (8). Its incidence appears to be relatively higher in East Asia (9). PB is thought to arise from multipotent stem cells, as evidenced by the presence of endocrine components, acinar cells with zymogen granules, and elevated AFP levels (10). Approximately 25%–55% of PB patients exhibit elevated AFP levels (11). The primary symptoms reported were abdominal pain and a palpable abdominal mass, occasionally accompanied by anorexia, diarrhea, and fever. In cases where the tumor compresses the biliary system, obstructive jaundice may occur.

Imaging studies play a crucial role in the initial diagnosis and evaluation of PB, particularly in assessing tumor characteristics and the extent of invasion. Previous reports indicate that PB typically appears as a heterogeneous or predominantly hypoechoic mass on US, sometimes containing small fluid-filled areas (9). In our two cases, one patient presented with a hypoechoic mass, whereas the other had a heterogeneous mass. Remarkably, one of our patients underwent CEUS, which showed arterial-phase hyperenhancement of solid components with persistent non-enhancing necrotic regions, followed by rapid venous-phase washout. There have been no reports of CEUS findings in pediatric PB except one case with adult PB who showed equal enhancement during the arterial phase and slightly faster washout in the delayed phase (12), which was consistent with our finding. It is recommended that CEUS be performed immediately after the detection of a pancreatic lesion provided superior lesion perfusion, contrast, and spatial resolution, in order to significantly enhance the accuracy of initial diagnostic imaging. The particular advantage of CEUS in the pediatric population lies in its dynamic assessment of microvascular perfusion in real time, without exposing the child to ionizing radiation. Unlike the fixed phases of CT and magnetic resonance imaging (MRI), CEUS allows continuous observation of contrast uptake and washout patterns, which may provide unique hemodynamic information. In these presented cases, the rapid washout pattern observed in CEUS contributed key diagnostic information. Therefore, rather than replacing these modalities, CEUS serves as a valuable complementary tool—particularly in specific clinical scenarios such as initial evaluation, follow-up of known lesions, or when other imaging findings are equivocal. Future studies directly comparing the diagnostic performance of these modalities in pediatric pancreatic tumors would be of significant clinical value.

The incidence of PB is very low, it often needs to be differentiated from other four main types of pancreatic tumors in children: solid pseudopapillary neoplasm (SPN), islet cell tumor, acinar cell carcinoma (ACC), and ductal adenocarcinoma (DAC) (1). SPN is currently regarded as the most common pancreatic tumor in children (2). It predominantly occurs in older children, particularly among adolescent women (13). On grayscale US, SPN typically presents as a well-defined, regular-shaped solid mass with a capsule, whereas on CEUS, it characteristically demonstrates heterogeneous enhancement with peripheral capsule enhancement and internal necrotic non-enhancement areas (14). Although SPN of the pancreas is classified as a low-grade malignancy, distant metastases and venous thrombosis are uncommon. A key differentiating feature is the serum AFP level: While SPN typically presents with normal AFP, elevated AFP is frequently observed in PB and serves as an important diagnostic clue (13). US and other imaging findings of PB include irregular morphology, clear boundaries, solid composition, and minimal evidence of large cystic areas or necrosis with heterogeneous hyperenhancement in the arterial phase and hypoenhancement in the venous phase. Additionally, PB may present secondary complications such as liver metastasis and venous thrombosis, one of two cases admitted to our hospital exhibited related secondary changes identified through intraoperative US.

Islet cell tumors are a type of functioning neuroendocrine tumor that typically occur in adults aged 40 to 50, often presenting with refractory hypoglycemia in children, so they are detected early. The size of mass is usually small, and it is easy to diagnose correctly depending on hypoglycemia in laboratory examinations, a conclusion further supported by its characteristic hypervascular pattern on CEUS, typically demonstrating rapid hyperenhancement in the arterial phase followed by washout (15). Approximately 15% of pediatric pancreatic tumors are ACCs (16), although cases of pancreatic ACCs in children are rare (17). Differentiating ACC from PB can be challenging due to their overlapping morphological and immunohistochemical features (18). The ability of radiomics to capture subtle tissue heterogeneity through texture and higher-order features offers a powerful tool for distinguishing phenotypically similar pancreatic tumors (19, 20). For instance, CT-based radiomic models can differentiate pancreatic neuroendocrine tumors from DAC with high accuracy (AUC 0.86–0.99) (20). This principle could potentially aid in refining SPN and PB differentiation by quantifying texture or enhancement differences. DACs are more common in individuals over 60 years of age and are exceedingly rare in children (21). On CEUS, DAC typically exhibits hypoenhancement in all phases due to its low vascular density (15). In addition to the above differential diagnosis, pediatric pancreatic lymphoma also needs to be differentiated with PB, consisting of less than 0.5% of pancreatic cancers (22). AFP levels in pancreatic lymphoma generally remain within the normal limits. On ultrasound, pancreatic lymphoma typically appears as a homogeneous hypoechoic mass confined to the pancreas (22). Lymphoma frequently involves multiple sites. Further examinations of retroperitoneal and superficial lymph enlargement, intestinal wall, liver, and spleen lesions suggest the possibility of lymphoma, but the final diagnosis still needs pathological examination. In summary, the differentiation of pediatric pancreatic lesions—with PB as the central focus—should be approached comprehensively, taking into account incidence, clinical presentation, laboratory markers, and ultrasound imaging features. To facilitate a clearer and more concise comparison, the key information is summarized in the table (**Table 1**) below.

**Table 1:** Summary of differential diagnosis for pediatric pancreatic lesions.

Lesion type	Predilection population	Clinical features	Laboratory tests	B-mode US	CEUS pattern
Pancreatoblastoma	Children	Associated with liver metastasis and venous thrombosis	AFP often elevated	Irregular solid mass, clear margin, few cystic areas	Heterogeneous hyperenhancement in arterial phase, hypoenhancement in venous phase
Solid pseudopapillary neoplasm	Adolescent women (most common)	Low-grade malignancy; distant metastases are uncommon	AFP normal	Well-circumscribed, encapsulated solid mass	Heterogeneous enhancement with peripheral capsule enhancement and necrotic areas
Islet cell tumor	Most common in adults 40-50	Whipple’s triad	Hypoglycemia Hyperinsulinemia	Small solid nodule	Arterial phase hyperenhancement with late-phase washout
Acinar cell carcinoma	Children (rare)	–	–	Morphology overlaps PB	Overlaps PB
Ductal adenocarcinoma	>60 years old (very rare in children)	–	–	Hypoechoic solid mass	Hypo-enhancement in all phases (hypo-vascular)
Pancreatic lymphoma	Children (very rare, <0.5%)	Often involves multiple sites (e.g., lymph nodes, liver, spleen)	AFP normal	Homogeneous hypoechoic mass	–

The final definitive diagnosis of PB relies on histopathology. Histological findings include the presence of characteristic squamous corpuscles and tumor cells with acinar, glandular, or undifferentiated features (3). Complete tumor resection is a key factor influencing prognosis in children with PB. For patients in whom surgical resection is not feasible, preoperative chemotherapy is recommended to reduce tumor size and create conditions favorable for surgery. Additionally, appropriate postoperative chemotherapy can help prevent tumor recurrence and prolong survival in cases of incomplete tumor resection (1). In our two cases, intraoperative ultrasound revealed a portal vein cancer thrombus, directly influencing the surgical approach. As a simple bedside imaging modality, intraoperative US provides surgeons with real-time information about lesions, as well as the extent of malignant tumor invasion into surrounding tissues and blood vessels. Therefore, intraoperative US should be performed as much as possible during surgery in PB children as an important addition to routine imaging examinations in order to ensure the surgery effectiveness and the best prognosis. This aligns with the broader role of intraoperative imaging guidance in minimally invasive pancreatic surgery (MIPS), where real-time anatomical visualization is critical for assessing resectability and preventing vascular injury (23). While PB surgery is frequently performed via open approaches given its rarity in children and potential for vascular involvement, the principles of intraoperative imaging, such as confirming tumor margins or vascular invasion, remain equally relevant (19).

In summary, accurately diagnosing PB prior to surgery remains a significant challenge. While PB lacks specific imaging characteristics, imaging can still provide clinicians with critical information regarding the tumor’s location, size, nature, extent of invasion, and blood supply, which aids in early and accurate diagnosis and treatment. Compared with 2D gray-scale US, CEUS offers more comprehensive and detailed information of lesions, enhancing the likelihood and accuracy of early diagnosis. Intraoperative US can provide real-time insights about the extent of malignant tumor invasion, assisting surgeons in making intraoperative decisions to ultimately achieving a better prognosis. It is important to note that the present report is limited to two cases, which curtails the generalizability of our sonographic observations. Future efforts should focus on large-scale, multicenter collaborations to corroborate the described imaging features of pediatric PB and to definitively establish the value of CEUS in standard diagnostic protocols.

## Data Availability

The raw data supporting the conclusions of this article will be made available by the authors, without undue reservation.
